# Effects of Oxidative Stress on Mesenchymal Stem Cell Biology

**DOI:** 10.1155/2016/2989076

**Published:** 2016-06-16

**Authors:** Ryan A. Denu, Peiman Hematti

**Affiliations:** ^1^Medical Scientist Training Program, School of Medicine and Public Health, University of Wisconsin-Madison, Madison, WI 53705, USA; ^2^Department of Medicine, Hematology/Oncology Division, University of Wisconsin-Madison, School of Medicine and Public Health, Madison, WI 53705, USA; ^3^University of Wisconsin Carbone Cancer Center, Madison, WI 53705, USA

## Abstract

Mesenchymal stromal/stem cells (MSCs) are multipotent stem cells present in most fetal and adult tissues.* Ex vivo* culture-expanded MSCs are being investigated for tissue repair and immune modulation, but their full clinical potential is far from realization. Here we review the role of oxidative stress in MSC biology, as their longevity and functions are affected by oxidative stress. In general, increased reactive oxygen species (ROS) inhibit MSC proliferation, increase senescence, enhance adipogenic but reduce osteogenic differentiation, and inhibit MSC immunomodulation. Furthermore, aging, senescence, and oxidative stress reduce their* ex vivo* expansion, which is critical for their clinical applications. Modulation of sirtuin expression and activity may represent a method to reduce oxidative stress in MSCs. These findings have important implications in the clinical utility of MSCs for degenerative and immunological based conditions. Further study of oxidative stress in MSCs is imperative in order to enhance MSC* ex vivo* expansion and* in vivo* engraftment, function, and longevity.

## 1. Introduction

Mesenchymal stromal/stem cells (MSCs) are multipotent cells characterized by their ability to differentiate into adipocytes, chondrocytes, and osteoblasts, their expression of surface markers CD73, CD90, and CD105, and their lack of hematopoietic lineage markers [1–4]. MSCs were initially studied for their ability to support hematopoietic stem cells in the bone marrow, but now they are being studied for their regenerative and immunomodulatory properties, as they home to injured tissues and contribute to tissue repair and suppression of inflammatory damage [5, 6]. MSCs have been isolated from a number of different tissues, including bone marrow, adipose, heart, vocal cord, and pancreatic islets [7–10]. They are also present in the tumor microenvironment, where they support the growth of tumor cells, activate mitogen and stress signaling, and increase resistance to cytotoxins [11–13].

MSCs have immunomodulatory properties and suppress the proliferation of CD4^+^ T cells, CD8^+^ T cells, B cells, and NK cells, while they induce the proliferation of regulatory T cells (Tregs) [5, 6, 14–21]. In addition, MSCs alternatively activate macrophages and bias them toward an immunosuppressive M2 phenotype [22]. Further evidence of MSCs creating a more anti-inflammatory state includes the following actions: induction of type 1 dendritic cells to reduce TNF*α* secretion and type 2 dendritic cells to increase IL-10 secretion [16, 23, 24]; causing Th1 cells to decrease IFN*γ* secretion and Th2 cells to increase IL-4 secretion [16]; decreasing NK cell proliferation and IFN*γ* secretion [14]; and converting macrophages to an anti-inflammatory immunophenotype [22]. At the same time, MSCs express low levels of MHC class I and no MHC class 2 and costimulatory molecules CD40, CD80, and CD86, preventing alloreactive antibody production and destruction [25, 26]. Due to these multimodal properties, MSCs are being studied for their potential use in different modes of therapy: (1) produce new tissues (e.g., cartilage repair); (2) assist with healing tissue damage (e.g., cardiovascular disease); (3) improve engraftment of other cells and tissues (e.g., hematopoietic cells and pancreatic islets); and (4) treat immune based pathologies (e.g., graft versus host disease, GVHD) [27–37].

MSCs have also been extensively studied because of their ability to differentiate into adipocytes, chondrocytes, and osteoblasts, which has significant potential in the field of regenerative medicine. However, MSCs are much farther from reaching clinical utility in regenerative medicine as compared to their utility in immunomodulation. Their chondrogenic ability has arguably gained the most attention [38] and could be utilized to aid in reconstitution of connective tissue loss in many joints, namely, the knee, which is crucial given the fact that chondrocytes are terminally differentiated, quiescent cells and do not regenerate damaged tissue.

While MSCs have been utilized with some success in the clinic, there is room for improvement in order for them to reach their full clinical potential. First, MSCs are rare cells* in situ* and must be expanded* ex vivo* in order to be utilized in the clinic. However, MSCs undergo replicative senescence, limiting the number of divisions [39–41]. Furthermore, this replicative senescence also compromises their immunomodulatory and differentiation functions and possibly their clinical activity against GVHD and other inflammatory pathologies [42, 43]. In addition, there is a lack of a well-defined and accepted potency assay to functionally assess MSC products [37, 44].

Another problem is the loss of transplanted MSCs at the site of graft, particularly after* ex vivo* culture [45, 46], which could possibly be due to loss of chemokine receptors [47]. Reactive oxygen species (ROS) and nonspecific inflammation generated at the ischemic site of injury have been hypothesized to lead to loss of transplanted MSCs from this site [48–50]. Therefore, there is great need to identify methods to manipulate MSCs to reduce ROS in both the MSCs themselves during their culture expansion production phase and in the injured tissue microenvironment in order to promote MSC engraftment and enhance tissue repair. First, this requires an understanding of the contributions of oxidative stress to MSC biology.

## 2. Oxidative Stress and MSC Differentiation

Oxidative stress is characterized by deregulated production and/or scavenging of reactive oxygen and nitrogen species (ROS and RNS, resp.). ROS are primarily generated from mitochondrial complexes I and III and NADPH oxidase isoform NOX4 during MSC differentiation [51]. The accumulation of free radicals can damage essentially all biomolecules, including DNA, protein, and lipids. High ROS levels cause cellular damage and dysfunction, but it is thought that a low basal level of ROS is necessary and advantageous in order to maintain cellular proliferation, differentiation, and survival [52–54]. Indeed, at baseline, MSCs have low levels of ROS and high levels of glutathione, the major cellular antioxidant [55]; however, other reports suggest that MSCs have low antioxidant activity and are more sensitive to oxidative stress compared to more differentiated cell types [56, 57]. In MSCs, excess ROS or exogenous addition of H_2_O_2_ can impair self-renewal, differentiation capacity, and proliferation [57–61]; concordantly, antioxidants stimulate MSC proliferation [62].

With regard to osteogenic differentiation, most studies suggest that ROS inhibit osteogenic differentiation [63]. Furthermore, addition of exogenous H_2_O_2_ reduces osteogenic differentiation in human and murine MSCs and osteoblast precursors [63–65]. In addition, MSCs from older donors demonstrate decreased osteogenic potential [66].* In vitro* induction of osteogenesis in human MSCs is associated with an upregulation of mtDNA copy number, protein subunits of respiratory enzymes, superoxide dismutase 2 (SOD2, alias MnSOD), catalase oxygen consumption rate, and antioxidant enzymes, but a decrease in ROS [63]; undifferentiated MSCs showed higher levels of glycolytic enzymes and a higher lactate production rate, suggesting that MSCs rely more on glycolysis for energy supply in comparison with MSC-differentiated osteoblasts, which rely more on oxidative mitochondrial metabolism. These findings support the idea that ROS and oxidative stress must decrease to allow for osteogenic differentiation to proceed. However, it appears that at least a basal level of ROS may be required, as some reports show that ROS enhance calcification and osteogenesis [67]; one caveat is that this study investigated murine vascular smooth muscle cells, which could explain the difference. In summary, ROS and aging inhibit MSC osteogenesis.

With regard to adipogenesis, ROS increase as MSCs differentiate into adipocytes, but it is unclear whether this is a cause or consequence of adipogenesis. Antioxidant enzymes such as SOD, catalase, and GPX are upregulated during adipogenesis in human MSCs [68]. It has been reported that ROS and the addition of exogenous H_2_O_2_ induce adipogenesis in human and murine MSCs and adipocyte precursors [51, 68, 69], lending credence for the idea that ROS play a causal role in adipogenesis. Furthermore, this effect of H_2_O_2_ is dose-dependent, as higher doses of H_2_O_2_ increased adipogenesis [70]. Consistent with ROS stimulating adipogenesis, the ROS scavenger* N*-acetylcysteine (NAC) inhibited adipogenesis in the mouse MSC cell line 10T1/2 [71]. In addition, it has been demonstrated that ROS generated by mitochondrial complex III are imperative for the activation of adipogenic transcription factors [72]. Similar to osteogenic differentiation, mitochondrial biogenesis and oxygen consumption increase significantly during adipogenesis [73, 74]. Additionally, inhibiting mitochondrial respiration significantly suppresses adipogenic differentiation [73], which makes sense for two reasons: (1) mitochondrial biogenesis and metabolism are thought to be important for MSC differentiation [74] and (2) inhibiting mitochondrial metabolism reduces ROS, and ROS are thought to stimulate adipogenesis. There is an increase in SOD3 expression with the differentiation of human MSCs into adipocytes [75] and during the early stages of adipogenic differentiation in 3T3-L1 cells [54]. However, there are some reports that contradict the idea that ROS stimulate adipogenesis; these reports demonstrate that aging and senescence, which are often associated with higher oxidative stress, decrease adipogenic differentiation [41, 76–78]. Additionally, RNAi-mediated depletion of MnSOD, which results in higher ROS, reduces the expression of late adipogenesis markers such as adiponectin and fatty acid-binding protein 4 (FABP4) [79]. Nevertheless, the prevailing view is that ROS and aging enhance adipogenesis [54].

ROS generally increase during chondrogenesis, and ROS generated by NADPH oxidases 2 and 4 are necessary for chondrogenic differentiation of murine primary chondrocytes and the ATDC5 cell line [80]. Consistent with this, SOD3 levels were reduced upon chondrogenesis [75]; SOD3 is known to help reduce ROS in the extracellular matrix. Furthermore, ROS scavenging with NAC blocked chondrogenic differentiation [80]. Consistent with this, increasing ROS levels stimulated chondrocyte hypertrophy, and this effect was inhibited by NAC [81].

## 3. Oxidative Stress and MSC Immunomodulation

Evidence of the direct role of oxidative stress in MSC immunomodulation is lacking. However, we do know that as MSCs are expanded* ex vivo*, proliferation decreases, oxidative stress increases, the level of certain surface antigens decreases (e.g., CD13, CD29, and CD44), and the ability to suppress T cell proliferation diminishes [41, 82, 83]. Similarly, MSCs from older donors, which also likely have greater oxidative stress, have reduced capacity to inhibit T cell proliferation [84, 85]. In addition, MSCs from human patients with atherosclerosis and type 2 diabetes, two diseases associated with elevated oxidative stress, have reduced ability to inhibit T cell proliferation [85]. However, some studies conflict with the assertion that donor age negatively impacts MSC suppression of T cell proliferation [86, 87]; one of these studies analyzed 53 human donors ranging within 13–80 years demonstrated no significant correlation between age and T cell suppression capability [86].

As most of the clinical uses of MSCs are dependent on their immunomodulatory properties, it will be important to continue to elucidate how oxidative stress affects MSC immunomodulation and whether or not modulating ROS and oxidative stress can enhance MSC* ex vivo* expansion, immunomodulation, and clinical utility.

## 4. Sirtuins, Oxidative Stress, and* Ex Vivo* Expansion of MSCs

Oxidative stress also affects* ex vivo* culture expansion and longevity of MSCs, which has implications for cell therapy. As MSCs are continuously passaged and grown* ex vivo*, they undergo replicative senescence, and proliferation decreases [39–42, 88, 89]. Aging and senescence are associated with greater oxidative stress, which limit the number of times that MSCs can be passaged and the quality of the cells [90, 91]. Therefore, there is great need to identify methods to prevent oxidative stress and replicative senescence in MSCs.

One potential method to reduce oxidative stress in MSCs is by modulating sirtuin expression and/or activity. Sirtuins are protein deacetylases that are thought to play evolutionarily conserved roles in lifespan extension [92–94]. Humans have seven sirtuins (SIRT1–7) that localize to distinct subcellular compartments and serve very distinct functions [95, 96]. In general, sirtuins are protective against age-related pathologies such as hearing loss [97], neurodegeneration [98], metabolic disease [99, 100], and cancer [101, 102]. Their roles in MSCs have not been fully elucidated and represent an interesting avenue of future research.

SIRT1, SIRT6, and SIRT7 localize to the nucleus. SIRT1 deacetylates a number of protein substrates including p53, DNA methyltransferase 1 (DNMT1), NF-*κ*B, forkhead transcription factors, PGC-1*α*, and histones [103–108]; an unbiased mass spectrometry-based acetylome analysis has revealed many more potential substrates [109]. SIRT1 knockdown decreases MSC proliferation and differentiation and increases senescence, and the opposite occurs with SIRT1 overexpression [110]. Consistently, SIRT1 activation with resveratrol enhances MSC osteogenesis [111]. SIRT1 is downregulated during human embryonic stem cell (ESC) differentiation at both mRNA and protein levels [112]. Therefore, SIRT1 is crucial for stem cell maintenance and differentiation. SIRT6 deacetylates histones H3K9Ac and H3K56Ac [113, 114] and is an imperative regulator of metabolism, transcription, telomere maintenance, and DNA repair in response to oxidative stress [115, 116]. It ADP-ribosylates and thereby activates PARP1, allowing for efficient double-strand break repair in the face of oxidative stress [116]. Furthermore, SIRT6 rescues the decline of base excision repair and homologous recombination repair during replicative senescence in primary human fibroblast strains [117, 118]. A study of human dermal fibroblasts from older subjects demonstrated that reprogramming into induced pluripotent stem cells (iPSCs) with Yamanaka factors was less efficient than in fibroblasts from older subjects, but that adding SIRT6 improved the efficiency of reprogramming [119]. Specific to MSCs, knockdown of SIRT6 inhibited while overexpression enhanced osteogenesis in rat MSCs [120]. Recent studies have demonstrated the possibility of activating SIRT6 with long-chain fatty acids [121], which may represent a way to modulate MSC function and longevity. The final nuclear sirtuin, SIRT7, has been less well characterized but localizes to the nucleolus and regulates rDNA transcription [122]. This is dependent on the deacetylation of U3-55k, a component of the U3 snoRNP complex, and this deacetylation enhances rRNA transcription and processing [123]. It is also important for proliferation and inhibition of apoptosis [122], perhaps via deacetylation of p53 [124]. In addition, SIRT7 has been recently shown to promote the regenerate capacity of aged hematopoietic stem cells, as inactivation increased mitochondrial protein folding stress and reduced regenerative capacity [125].

SIRT3, SIRT4, and SIRT5 localize to the mitochondria, where approximately 90% of ROS are produced in mitochondria [126]. SIRT3 is the major mitochondrial deacetylase and reprograms mitochondrial metabolism away from carbohydrate metabolism in favor of more efficient electron transport, which is thought to result in reduced ROS production [127–130]. SIRT3 deacetylates and thereby activates isocitrate dehydrogenase 2 (IDH2), an enzyme that catalyzes the TCA cycle redox conversion of isocitrate to *α*-ketoglutarate and serves as a major source of NADPH production [97, 131, 132]. SIRT3 also deacetylates and thereby activates superoxide dismutase 2 (SOD2, alias MnSOD), which also neutralizes ROS [133–135]. One of the only studies of SIRT3 on stem cell function demonstrated that SIRT3 is not required for hematopoietic stem cell (HSC) maintenance and tissue homeostasis at a young age in mice; however, SIRT3 is imperative in HSCs at an older age and under stress [136]. Importantly, SIRT3 expression decreases with aging, and this is accompanied by a concomitant decrease in SOD2 activity; overexpressing SIRT3 in these aged HSCs reduces oxidative stress and improves their regenerative capacity [136]. SIRT3 overexpression can also protect against low-oxygen and low-glucose stresses [137]. Similarly, SOD2 acetylation, a target of SIRT3 deacetylation, increases with age in rats and humans, which can be restored* in vitro* by adding recombinant SIRT3 [138]. The next mitochondrial sirtuin, SIRT4, mono-ADP-ribosylates and thereby inhibits glutamate dehydrogenase, which slows the conversion of glutamate to *α*-ketoglutarate [139–141]. SIRT4 is also a lipoamidase that hydrolyzes the lipoamide cofactors from the E2 component of the pyruvate dehydrogenase (PDH) complex, which reduces the activity of the complex [142]. Defects in the PDH complex have been shown to increase ROS and oxidative stress [143]. Similarly, SIRT4 and ROS are upregulated during replicative senescence and in response to DNA damage [144, 145]; however, one study shows that SIRT4 depletion reduces ROS [144] and therefore suggests that inhibiting SIRT4 may be a strategy to prevent oxidative stress in MSCs, while another believes that SIRT4 is required for appropriate recovery from cellular stresses [145]. Further study of SIRT4 in MSCs is warranted. The final mitochondrial sirtuin, SIRT5, is the major mitochondrial desuccinylase [146, 147]. It desuccinylates and activates SOD1 to facilitate the elimination of ROS [148]. SIRT5 has not been studied in stem cells, but based on these known functions, we hypothesize that SIRT5 helps maintain ROS at low levels to preserve stem cell function and longevity.

SIRT2 is a cytoplasmic sirtuin that deacetylates and therefore destabilizes *α*-tubulin [149]. Furthermore, SIRT2 deacetylates p300, a histone acetyltransferase crucial for many biological processes including cellular proliferation and differentiation, which increases the affinity of p300 for preinitation complexes [150]; in this way, SIRT2 may help control transcription of genes needed for MSC differentiation. SIRT2 gene expression increases with differentiation of mouse ESCs [151]. In stem cells, SIRT2 has been shown to inhibit the expression of keratin 19, which is a stem cell marker [152]. These two studies suggest that SIRT2 is more important for differentiation. However, it may be specific to the type of differentiation being discussed; downregulation of SIRT2 promotes 3T3L1 adipocyte differentiation [153], and SIRT2 knockdown in mouse ESCs promotes differentiation into mesodermal and endodermal tissues and diminishes differentiation into ectodermal tissues [151]. Lastly, SIRT2 may play a role in autophagy, the catabolic process that allows the cell to recycle damaged proteins and organelles and has been shown to be important for stem cell function, particularly in the face of oxidative stress [154]; however, more work needs to be done here, as the exact role of autophagy in MSC biology is unclear. Nevertheless, most reports suggest that SIRT2 inhibits autophagy. One report shows that depletion of SIRT2 activates autophagy [155]. Consistent with this, another study shows that the FoxO1 transcription factor is required for autophagy caused by oxidative stress, and that dissociation from SIRT2 increases FoxO1 acetylation and induction of autophagy [156]; this suggests that SIRT2 inhibits autophagy. Therefore, modulation of SIRT2 expression and activity is worth further pursuing.

## 5. Conclusions

MSCs have immense therapeutic potential; yet this potential has not been reached for a number of reasons. Perhaps one of these reasons is the effect of oxidative stress on MSC* ex vivo* expansion, leading to problems with* in vivo* function and engraftment. Therefore, there is great need to identify novel methods to optimize ROS levels in MSCs to enhance their immunomodulatory and regenerative abilities so that their full therapeutic potential can be realized. The sirtuins represent a potential way to achieve this and warrant further study in MSCs. Many of the sirtuins may help enhance our ability to expand MSCs* ex vivo* for eventual clinical use.

## Abbreviations

ESC: Embryonic stem cell
IFN*γ*: Interferon gamma
iPSC: Induced pluripotent stem cell
MSC: Mesenchymal stromal/stem cell
GVHD: Graft versus host disease
NAC: * N*-Acetylcysteine
PPAR*γ*: Peroxisome proliferator-activated receptor gamma
RNS: Reactive nitrogen species
ROS: Reactive oxygen species
SIRT: Sirtuin
SOD: Superoxide dismutase.

## References

[1] Dominici M., Le Blanc K., Mueller I. (2006). Minimal criteria for defining multipotent mesenchymal stromal cells. The International Society for Cellular Therapy position statement. *Cytotherapy*.

[2] Friedenstein A. J., Petrakova K. V., Kurolesova A. I., Frolova G. P. (1968). Heterotopic of bone marrow. Analysis of precursor cells for osteogenic and hematopoietic tissues. *Transplantation*.

[3] Pittenger M. F., Mackay A. M., Beck S. C. (1999). Multilineage potential of adult human mesenchymal stem cells. *Science*.

[4] Denu R. A., Nemcek S., Bloom D. D. (2016). Fibroblasts and mesenchymal stromal/stem cells are phenotypically indistinguishable. *Acta Haematologica*.

[5] Nicola M. D., Carlo-Stella C., Magni M. (2002). Human bone marrow stromal cells suppress T-lymphocyte proliferation induced by cellular or nonspecific mitogenic stimuli. *Blood*.

[6] Bartholomew A., Sturgeon C., Siatskas M. (2002). Mesenchymal stem cells suppress lymphocyte proliferation in vitro and prolong skin graft survival in vivo. *Experimental Hematology*.

[7] Kim J., Breunig M. J., Escalante L. E. (2012). Biologic and immunomodulatory properties of mesenchymal stromal cells derived from human pancreatic islets. *Cytotherapy*.

[8] Hanson S. E., Kim J., Quinchia Johnson B. H. (2010). Characterization of mesenchymal stem cells from human vocal fold fibroblasts. *Laryngoscope*.

[9] Lushaj E. B., Anstadt E., Haworth R. (2011). Mesenchymal stromal cells are present in the heart and promote growth of adult stem cells in vitro. *Cytotherapy*.

[10] Hanson S. E., Kim J., Hematti P. (2013). Comparative analysis of adipose-derived mesenchymal stem cells isolated from abdominal and breast tissue. *Aesthetic Surgery Journal*.

[11] Castellone M. D., Laatikainen L. E., Laurila J. P. (2013). Brief report: mesenchymal stromal cell atrophy in coculture increases aggressiveness of transformed cells. *Stem Cells*.

[12] Kim J., Denu R. A., Dollar B. A. (2012). Macrophages and mesenchymal stromal cells support survival and proliferation of multiple myeloma cells. *British Journal of Haematology*.

[13] Cammarota F., Laukkanen M. O. (2016). Mesenchymal stem/stromal cells in stromal evolution and cancer progression. *Stem Cells International*.

[14] Spaggiari G. M., Capobianco A., Becchetti S., Mingari M. C., Moretta L. (2006). Mesenchymal stem cell-natural killer cell interactions: evidence that activated NK cells are capable of killing MSCs, whereas MSCs can inhibit IL-2-induced NK-cell proliferation. *Blood*.

[15] English K., Ryan J. M., Tobin L., Murphy M. J., Barry F. P., Mahon B. P. (2009). Cell contact, prostaglandin E2 and transforming growth factor beta 1 play non-redundant roles in human mesenchymal stem cell induction of CD4+CD25Highforkhead box P3+ regulatory T cells. *Clinical and Experimental Immunology*.

[16] Aggarwal S., Pittenger M. F. (2005). Human mesenchymal stem cells modulate allogeneic immune cell responses. *Blood*.

[17] Krampera M., Glennie S., Dyson J. (2003). Bone marrow mesenchymal stem cells inhibit the response of naive and memory antigen-specific T cells to their cognate peptide. *Blood*.

[18] Corcione A., Benvenuto F., Ferretti E. (2006). Human mesenchymal stem cells modulate B-cell functions. *Blood*.

[19] Le Blanc K., Tammik L., Sundberg B., Haynesworth S. E., Ringdén O. (2003). Mesenchymal stem cells inhibit and stimulate mixed lymphocyte cultures and mitogenic responses independently of the major histocompatibility complex. *Scandinavian Journal of Immunology*.

[20] Le Blanc K., Ringdén O. (2007). Immunomodulation by mesenchymal stem cells and clinical experience. *Journal of Internal Medicine*.

[21] Bloom D. D., Centanni J. M., Bhatia N. (2015). A reproducible immunopotency assay to measure mesenchymal stromal cell-mediated T-cell suppression. *Cytotherapy*.

[22] Kim J., Hematti P. (2009). Mesenchymal stem cell-educated macrophages: a novel type of alternatively activated macrophages. *Experimental Hematology*.

[23] Zhang W., Ge W., Li C. (2004). Effects of mesenchymal stem cells on differentiation, maturation, and function of human monocyte-derived dendritic cells. *Stem Cells and Development*.

[24] Zhang B., Liu R., Shi D. (2009). Mesenchymal stem cells induce mature dendritic cells into a novel Jagged-2 dependent regulatory dendritic cell population. *Blood*.

[25] Tse W. T., Pendleton J. D., Beyer W. M., Egalka M. C., Guinan E. C. (2003). Suppression of allogeneic T-cell proliferation by human marrow stromal cells: implications in transplantation. *Transplantation*.

[26] Sotiropoulou P. A., Perez S. A., Gritzapis A. D., Baxevanis C. N., Papamichail M. (2006). Interactions between human mesenchymal stem cells and natural killer cells. *Stem Cells*.

[27] Le Blanc K., Rasmusson I., Sundberg B. (2004). Treatment of severe acute graft-versus-host disease with third party haploidentical mesenchymal stem cells. *The Lancet*.

[28] Kunter U., Rong S., Djuric Z. (2006). Transplanted mesenchymal stem cells accelerate glomerular healing in experimental glomerulonephritis. *Journal of the American Society of Nephrology*.

[29] Gharibi T., Ahmadi M., Seyfizadeh N., Jadidi-Niaragh F., Yousefi M. (2015). Immunomodulatory characteristics of mesenchymal stem cells and their role in the treatment of multiple sclerosis. *Cellular Immunology*.

[30] Orlic D., Kajstura J., Chimenti S. (2001). Mobilized bone marrow cells repair the infarcted heart, improving function and survival. *Proceedings of the National Academy of Sciences of the United States of America*.

[31] Wyles C. C., Houdek M. T., Behfar A., Sierra R. J. (2015). Mesenchymal stem cell therapy for osteoarthritis: current perspectives. *Stem Cells and Cloning: Advances and Applications*.

[32] Volarevic V., Nurkovic J., Arsenijevic N., Stojkovic M. (2014). Concise review: therapeutic potential of mesenchymal stem cells for the treatment of acute liver failure and cirrhosis. *Stem Cells*.

[33] Ringdén O., Uzunel M., Rasmusson I. (2006). Mesenchymal stem cells for treatment of therapy-resistant graft-versus-host disease. *Transplantation*.

[34] Laurila J. P., Laatikainen L., Castellone M. D. (2009). Human embryonic stem cell-derived mesenchymal stromal cell transplantation in a rat hind limb injury model. *Cytotherapy*.

[35] Battiwalla M., Hematti P. (2009). Mesenchymal stem cells in hematopoietic stem cell transplantation. *Cytotherapy*.

[36] Hematti P., Kim J., Stein A. P., Kaufman D. (2013). Potential role of mesenchymal stromal cells in pancreatic islet transplantation. *Transplantation Reviews*.

[37] Hematti P. (2016). Characterization of mesenchymal stromal cells: potency assay development. *Transfusion*.

[38] Brown P. T., Handorf A. M., Jeon W. B., Li W.-J. (2013). Stem cell-based tissue engineering approaches for musculoskeletal regeneration. *Current Pharmaceutical Design*.

[39] Bajek A., Czerwinski M., Olkowska J., Gurtowska N., Kloskowski T., Drewa T. (2012). Does aging of mesenchymal stem cells limit their potential application in clinical practice?. *Aging Clinical and Experimental Research*.

[40] Lepperdinger G. (2011). Inflammation and mesenchymal stem cell aging. *Current Opinion in Immunology*.

[41] Wagner W., Horn P., Castoldi M. (2008). Replicative senescence of mesenchymal stem cells: a continuous and organized process. *PLoS ONE*.

[42] Banfi A., Muraglia A., Dozin B., Mastrogiacomo M., Cancedda R., Quarto R. (2000). Proliferation kinetics and differentiation potential of ex vivo expanded human bone marrow stromal cells: implications for their use in cell therapy. *Experimental Hematology*.

[43] von Bahr L., Sundberg B., Lönnies L. (2012). Long-term complications, immunologic effects, and role of passage for outcome in mesenchymal stromal cell therapy. *Biology of Blood and Marrow Transplantation*.

[44] Galipeau J., Krampera M., Barrett J. (2016). International Society for Cellular Therapy perspective on immune functional assays for mesenchymal stromal cells as potency release criterion for advanced phase clinical trials. *Cytotherapy*.

[45] Rombouts W. J. C., Ploemacher R. E. (2003). Primary murine MSC show highly efficient homing to the bone marrow but lose homing ability following culture. *Leukemia*.

[46] Galderisi U., Giordano A. (2014). The gap between the physiological and therapeutic roles of mesenchymal stem cells. *Medicinal Research Reviews*.

[47] Honczarenko M., Le Y., Swierkowski M., Ghiran I., Glodek A. M., Silberstein L. E. (2006). Human bone marrow stromal cells express a distinct set of biologically functional chemokine receptors. *Stem Cells*.

[48] Yang S.-R., Park J.-R., Kang K.-S. (2015). Reactive oxygen species in mesenchymal stem cell aging: implication to lung diseases. *Oxidative Medicine and Cellular Longevity*.

[49] Pittenger M. F., Martin B. J. (2004). Mesenchymal stem cells and their potential as cardiac therapeutics. *Circulation Research*.

[50] Devine S. M., Bartholomew A. M., Mahmud N. (2001). Mesenchymal stem cells are capable of homing to the bone marrow of non-human primates following systemic infusion. *Experimental Hematology*.

[51] Schröder K., Wandzioch K., Helmcke I., Brandes R. P. (2009). Nox4 acts as a switch between differentiation and proliferation in preadipocytes. *Arteriosclerosis, Thrombosis, and Vascular Biology*.

[52] Kobayashi C. I., Suda T. (2012). Regulation of reactive oxygen species in stem cells and cancer stem cells. *Journal of Cellular Physiology*.

[53] D'Autréaux B., Toledano M. B. (2007). ROS as signalling molecules: mechanisms that generate specificity in ROS homeostasis. *Nature Reviews Molecular Cell Biology*.

[54] Atashi F., Modarressi A., Pepper M. S. (2015). The role of reactive oxygen species in mesenchymal stem cell adipogenic and osteogenic differentiation: a review. *Stem Cells and Development*.

[55] Valle-Prieto A., Conget P. A. (2010). Human mesenchymal stem cells efficiently manage oxidative stress. *Stem Cells and Development*.

[56] Orciani M., Gorbi S., Benedetti M. (2010). Oxidative stress defense in human-skin-derived mesenchymal stem cells versus human keratinocytes: different mechanisms of protection and cell selection. *Free Radical Biology and Medicine*.

[57] Ko E., Lee K. Y., Hwang D. S. (2012). Human umbilical cord blood-derived mesenchymal stem cells undergo cellular senescence in response to oxidative stress. *Stem Cells and Development*.

[58] Meagher R. C., Salvado A. J., Wright D. G. (1988). An analysis of the multilineage production of human hematopoietic progenitors in long-term bone marrow culture: evidence that reactive oxygen intermediates derived from mature phagocytic cells have a role in limiting progenitor cell self-renewal. *Blood*.

[59] Ito K., Hirao A., Arai F. (2004). Regulation of oxidative stress by ATM is required for self-renewal of haematopoietic stem cells. *Nature*.

[60] Alves H., Munoz-Najar U., De Wit J. (2010). A link between the accumulation of DNA damage and loss of multi-potency of human mesenchymal stromal cells. *Journal of Cellular and Molecular Medicine*.

[61] Choo K. B. U., Tai L., Hymavathee K. S. (2014). Oxidative stress-induced premature senescence in Wharton's jelly-derived mesenchymal stem cells. *International Journal of Medical Sciences*.

[62] Zou X., Li H., Chen L., Baatrup A., Bünger C., Lind M. (2004). Stimulation of porcine bone marrow stromal cells by hyaluronan, dexamethasone and rhBMP-2. *Biomaterials*.

[63] Chen C.-T., Shih Y.-R. V., Kuo T. K., Lee O. K., Wei Y.-H. (2008). Coordinated changes of mitochondrial biogenesis and antioxidant enzymes during osteogenic differentiation of human mesenchymal stem cells. *Stem Cells*.

[64] Mody N., Parhami F., Sarafian T. A., Demer L. L. (2001). Oxidative stress modulates osteoblastic differentiation of vascular and bone cells. *Free Radical Biology and Medicine*.

[65] Almeida M., Han L., Martin-Millan M., O'Brien C. A., Manolagas S. C. (2007). Oxidative stress antagonizes Wnt signaling in osteoblast precursors by diverting *β*-catenin from T cell factor- to forkhead box O-mediated transcription. *The Journal of Biological Chemistry*.

[66] de Girolamo L., Lopa S., Arrigoni E., Sartori M. F., Baruffaldi Preis F. W., Brini A. T. (2009). Human adipose-derived stem cells isolated from young and elderly women: their differentiation potential and scaffold interaction during *in vitro* osteoblastic differentiation. *Cytotherapy*.

[67] Byon C. H., Javed A., Dai Q. (2008). Oxidative stress induces vascular calcification through modulation of the osteogenic transcription factor Runx2 by AKT signaling. *The Journal of Biological Chemistry*.

[68] Higuchi M., Dusting G. J., Peshavariya H. (2013). Differentiation of human adipose-derived stem cells into fat involves reactive oxygen species and forkhead box o1 mediated upregulation of antioxidant enzymes. *Stem Cells and Development*.

[69] Reykdal S., Abboud C., Liesveld J. (1999). Effect of nitric oxide production and oxygen tension on progenitor preservation in ex vivo culture. *Experimental Hematology*.

[70] Turker I., Zhang Y., Zhang Y., Rehman J. (2007). Oxidative stress as a regulator of adipogenesis. *The FASEB Journal*.

[71] Kanda Y., Hinata T., Kang S. W., Watanabe Y. (2011). Reactive oxygen species mediate adipocyte differentiation in mesenchymal stem cells. *Life Sciences*.

[72] Tormos K. V., Anso E., Hamanaka R. B. (2011). Mitochondrial complex III ROS regulate adipocyte differentiation. *Cell Metabolism*.

[73] Zhang Y., Marsboom G., Toth P. T., Rehman J. (2013). Mitochondrial respiration regulates adipogenic differentiation of human mesenchymal stem cells. *PLoS ONE*.

[74] Wilson-Fritch L., Burkart A., Bell G. (2003). Mitochondrial biogenesis and remodeling during adipogenesis and in response to the insulin sensitizer rosiglitazone. *Molecular and Cellular Biology*.

[75] Nightingale H., Kemp K., Gray E. (2012). Changes in expression of the antioxidant enzyme SOD3 occur upon differentiation of human bone marrow-derived mesenchymal stem cells in vitro. *Stem Cells and Development*.

[76] Bonab M. M., Alimoghaddam K., Talebian F., Ghaffari S. H., Ghavamzadeh A., Nikbin B. (2006). Aging of mesenchymal stem cell in vitro. *BMC Cell Biology*.

[77] Digirolamo C. M., Stokes D., Colter D., Phinney D. G., Class R., Prockop D. J. (1999). Propagation and senescence of human marrow stromal cells in culture: a simple colony-forming assay identifies samples with the greatest potential to propagate and differentiate. *British Journal of Haematology*.

[78] Alt E. U., Senst C., Murthy S. N. (2012). Aging alters tissue resident mesenchymal stem cell properties. *Stem Cell Research*.

[79] Krautbauer S., Eisinger K., Hader Y., Neumeier M., Buechler C. (2014). Manganese superoxide dismutase knock-down in 3T3-L1 preadipocytes impairs subsequent adipogenesis. *Molecular and Cellular Biochemistry*.

[80] Kim S. K., Choi H. W., Yoon H. E., Kim I. Y. (2010). Reactive oxygen species generated by NADPH oxidase 2 and 4 are required for chondrogenic differentiation. *The Journal of Biological Chemistry*.

[81] Morita K., Miyamoto T., Fujita N. (2007). Reactive oxygen species induce chondrocyte hypertrophy in endochondral ossification. *The Journal of Experimental Medicine*.

[82] Zaim M., Karaman S., Cetin G., Isik S. (2012). Donor age and long-term culture affect differentiation and proliferation of human bone marrow mesenchymal stem cells. *Annals of Hematology*.

[83] Ren J., Stroncek D. F., Zhao Y. (2013). Intra-subject variability in human bone marrow stromal cell (BMSC) replicative senescence: molecular changes associated with BMSC senescence. *Stem Cell Research*.

[84] Wu L. W., Wang Y.-L., Christensen J. M. (2014). Donor age negatively affects the immunoregulatory properties of both adipose and bone marrow derived mesenchymal stem cells. *Transplant Immunology*.

[85] Kizilay Mancini O., Shum-Tim D., Stochaj U., Correa J. A., Colmegna I. (2015). Age, atherosclerosis and type 2 diabetes reduce human mesenchymal stromal cell-mediated T-cell suppression. *Stem Cell Research and Therapy*.

[86] Siegel G., Kluba T., Hermanutz-Klein U., Bieback K., Northoff H., Schäfer R. (2013). Phenotype, donor age and gender affect function of human bone marrow-derived mesenchymal stromal cells. *BMC Medicine*.

[87] Landgraf K., Brunauer R., Lepperdinger G., Grubeck-Loebenstein B. (2011). The suppressive effect of mesenchymal stromal cells on T cell proliferation is conserved in old age. *Transplant Immunology*.

[88] Baxter M. A., Wynn R. F., Jowitt S. N., Wraith J. E., Fairbairn L. J., Bellantuono I. (2004). Study of telomere length reveals rapid aging of human marrow stromal cells following in vitro expansion. *STEM CELLS*.

[89] Cakouros D., Isenmann S., Cooper L. (2012). Twist-1 induces Ezh2 recruitment regulating histone methylation along the Ink4A/Arf locus in mesenchymal stem cells. *Molecular and Cellular Biology*.

[90] Stolzing A., Jones E., McGonagle D., Scutt A. (2008). Age-related changes in human bone marrow-derived mesenchymal stem cells: consequences for cell therapies. *Mechanisms of Ageing and Development*.

[91] Terman A., Brunk U. T. (2006). Oxidative stress, accumulation of biological ‘garbage’, and aging. *Antioxidants and Redox Signaling*.

[92] Guarente L. (2007). Sirtuins in aging and disease. *Cold Spring Harbor Symposia on Quantitative Biology*.

[93] Denu J. M. (2005). The sir 2 family of protein deacetylases. *Current Opinion in Chemical Biology*.

[94] Milne J. C., Denu J. M. (2008). The Sirtuin family: therapeutic targets to treat diseases of aging. *Current Opinion in Chemical Biology*.

[95] Finkel T., Deng C.-X., Mostoslavsky R. (2009). Recent progress in the biology and physiology of sirtuins. *Nature*.

[96] Feldman J. L., Dittenhafer-Reed K. E., Denu J. M. (2012). Sirtuin catalysis and regulation. *The Journal of Biological Chemistry*.

[97] Someya S., Yu W., Hallows W. C. (2010). Sirt3 mediates reduction of oxidative damage and prevention of age-related hearing loss under caloric restriction. *Cell*.

[98] Kim D., Nguyen M. D., Dobbin M. M. (2007). SIRT1 deacetylase protects against neurodegeneration in models for Alzheimer's disease and amyotrophic lateral sclerosis. *The EMBO Journal*.

[99] Smith B. C., Hallows W. C., Denu J. M. (2008). Mechanisms and molecular probes of sirtuins. *Chemistry and Biology*.

[100] Guarente L. (2006). Sirtuins as potential targets for metabolic syndrome. *Nature*.

[101] Kim H.-S., Patel K., Muldoon-Jacobs K. (2010). Sirt3 is a mitochondria-localized tumor suppressor required for maintenance of mitochondrial integrity and metabolism during stress. *Cancer Cell*.

[102] Sebastián C., Zwaans B. M. M., Silberman D. M. (2012). The histone deacetylase SIRT6 Is a tumor suppressor that controls cancer metabolism. *Cell*.

[103] Yeung F., Hoberg J. E., Ramsey C. S. (2004). Modulation of NF-*κ*B-dependent transcription and cell survival by the SIRT1 deacetylase. *The EMBO Journal*.

[104] Luo J., Nikolaev A. Y., Imai S.-I. (2001). Negative control of p53 by Sir2*α* promotes cell survival under stress. *Cell*.

[105] Vaziri H., Dessain S. K., Eaton E. N. (2001). *hSIR*2^*SIRT*1^ functions as an NAD-dependent p53 deacetylase. *Cell*.

[106] Peng L., Yuan Z., Ling H. (2011). SIRT1 deacetylates the DNA methyltransferase 1 (DNMT1) protein and alters its activities. *Molecular and Cellular Biology*.

[107] Brunet A., Sweeney L. B., Sturgill J. F. (2004). Stress-dependent regulation of FOXO transcription factors by the SIRT1 deacetylase. *Science*.

[108] Imai S.-I., Armstrong C. M., Kaeberlein M., Guarente L. (2000). Transcriptional silencing and longevity protein Sir2 is an NAD-dependent histone deacetylase. *Nature*.

[109] Chen Y., Zhao W., Yang J. S. (2012). Quantitative acetylome analysis reveals the roles of SIRT1 in regulating diverse substrates and cellular pathways. *Molecular and Cellular Proteomics*.

[110] Yuan H.-F., Zhai C., Yan X.-L. (2012). SIRT1 is required for long-term growth of human mesenchymal stem cells. *Journal of Molecular Medicine*.

[111] Shakibaei M., Shayan P., Busch F. (2012). Resveratrol mediated modulation of sirt-1/Runx2 promotes osteogenic differentiation of mesenchymal stem cells: potential role of Runx2 deacetylation. *PLoS ONE*.

[112] Calvanese V., Lara E., Suárez-Álvarez B. (2010). Sirtuin 1 regulation of developmental genes during differentiation of stem cells. *Proceedings of the National Academy of Sciences of the United States of America*.

[113] Michishita E., McCord R. A., Berber E. (2008). SIRT6 is a histone H3 lysine 9 deacetylase that modulates telomeric chromatin. *Nature*.

[114] Michishita E., McCord R. A., Boxer L. D. (2009). Cell cycle-dependent deacetylation of telomeric histone H3 lysine K56 by human SIRT6. *Cell Cycle*.

[115] Van Meter M., Mao Z., Gorbunova V., Seluanov A. (2011). Repairing split ends: SIRT6, mono-ADP ribosylation and DNA repair. *Aging*.

[116] Mao Z., Hine C., Tian X. (2011). SIRT6 promotes DNA repair under stress by activating PARP1. *Science*.

[117] Mao Z., Tian X., Van Meter M., Ke Z., Gorbunova V., Seluanov A. (2012). Sirtuin 6 (SIRT6) rescues the decline of homologous recombination repair during replicative senescence. *Proceedings of the National Academy of Sciences of the United States of America*.

[118] Xu Z., Zhang L., Zhang W. (2015). SIRT6 rescues the age related decline in base excision repair in a PARP1-dependent manner. *Cell Cycle*.

[119] Sharma A., Diecke S., Zhang W. Y. (2013). The role of SIRT6 protein in aging and reprogramming of human induced pluripotent stem cells. *The Journal of Biological Chemistry*.

[120] Sun H., Wu Y., Fu D., Liu Y., Huang C. (2014). SIRT6 regulates osteogenic differentiation of rat bone marrow mesenchymal stem cells partially via suppressing the nuclear factor-*κ*B signaling pathway. *STEM CELLS*.

[121] Feldman J. L., Baeza J., Denu J. M. (2013). Activation of the protein deacetylase sirt6 by long-chain fatty acids and widespread deacylation by mammalian sirtuins. *The Journal of Biological Chemistry*.

[122] Ford E., Voit R., Liszt G., Magin C., Grummt I., Guarente L. (2006). Mammalian Sir2 homolog SIRT7 is an activator of RNA polymerase I transcription. *Genes and Development*.

[123] Chen S., Blank M. F., Iyer A. (2016). SIRT7-dependent deacetylation of the U3-55k protein controls pre-rRNA processing. *Nature Communications*.

[124] Vakhrusheva O., Smolka C., Gajawada P. (2008). Sirt7 increases stress resistance of cardiomyocytes and prevents apoptosis and inflammatory cardiomyopathy in mice. *Circulation Research*.

[125] Mohrin M., Shin J., Liu Y. (2015). A mitochondrial UPR-mediated metabolic checkpoint regulates hematopoietic stem cell aging. *Science*.

[126] Balaban R. S., Nemoto S., Finkel T. (2005). Mitochondria, oxidants, and aging. *Cell*.

[127] Hirschey M. D., Shimazu T., Goetzman E. (2010). SIRT3 regulates mitochondrial fatty-acid oxidation by reversible enzyme deacetylation. *Nature*.

[128] Yu W., Denu R. A., Krautkramer K. A. (2016). Loss of sirt3 provides growth advantage for B cell malignancies. *The Journal of Biological Chemistry*.

[129] Hebert A. S., Dittenhafer-Reed K. E., Yu W. (2013). Calorie restriction and SIRT3 trigger global reprogramming of the mitochondrial protein acetylome. *Molecular Cell*.

[130] Dittenhafer-Reed K. E., Richards A. L., Fan J. (2015). SIRT3 mediates multi-tissue coupling for metabolic fuel switching. *Cell Metabolism*.

[131] Schlicker C., Gertz M., Papatheodorou P., Kachholz B., Becker C. F. W., Steegborn C. (2008). Substrates and regulation mechanisms for the human mitochondrial sirtuins Sirt3 and Sirt5. *Journal of Molecular Biology*.

[132] Yu W., Dittenhafer-Reed K. E., Denu J. M. (2012). SIRT3 protein deacetylates isocitrate dehydrogenase 2 (IDH2) and regulates mitochondrial redox status. *The Journal of Biological Chemistry*.

[133] Tao R., Coleman M. C., Pennington J. D. (2010). Sirt3-mediated deacetylation of evolutionarily conserved lysine 122 regulates MnSOD activity in response to stress. *Molecular Cell*.

[134] Chen Y., Zhang J., Lin Y. (2011). Tumour suppressor SIRT3 deacetylates and activates manganese superoxide dismutase to scavenge ROS. *EMBO Reports*.

[135] Qiu X., Brown K., Hirschey M. D., Verdin E., Chen D. (2010). Calorie restriction reduces oxidative stress by SIRT3-mediated SOD2 activation. *Cell Metabolism*.

[136] Brown K., Xie S., Qiu X. (2013). SIRT3 reverses aging-associated degeneration. *Cell Reports*.

[137] Shulyakova N., Sidorova-Darmos E., Fong J., Zhang G., Mills L. R., Eubanks J. H. (2014). Over-expression of the Sirt3 sirtuin Protects neuronally differentiated PC12 Cells from degeneration induced by oxidative stress and trophic withdrawal. *Brain Research*.

[138] Fu Y., Kinter M., Hudson J. (2016). Aging promotes SIRT3-dependent cartilage SOD2 acetylation and osteoarthritis. *Arthritis & Rheumatology*.

[139] Ahuja N., Schwer B., Carobbio S. (2007). Regulation of insulin secretion by SIRT4, a mitochondrial ADP-ribosyltransferase. *Journal of Biological Chemistry*.

[140] Komlos D., Mann K. D., Zhuo Y. (2013). Glutamate dehydrogenase 1 and SIRT4 regulate glial development. *Glia*.

[141] Haigis M. C., Mostoslavsky R., Haigis K. M. (2006). SIRT4 inhibits glutamate dehydrogenase and opposes the effects of calorie restriction in pancreatic *β* cells. *Cell*.

[142] Mathias R. A., Greco T. M., Oberstein A. (2014). Sirtuin 4 is a lipoamidase regulating pyruvate dehydrogenase complex activity. *Cell*.

[143] Stacpoole P. W. (2012). The pyruvate dehydrogenase complex as a therapeutic target for age-related diseases. *Aging Cell*.

[144] Lang A., Grether-Beck S., Singh M. (2016). MicroRNA-15b regulates mitochondrial ROS production and the senescence-associated secretory phenotype through sirtuin 4/SIRT4. *Aging*.

[145] Jeong S. M., Hwang S., Seong R. H. (2016). SIRT4 regulates cancer cell survival and growth after stress. *Biochemical and Biophysical Research Communications*.

[146] Du J., Zhou Y., Su X. (2011). Sirt5 is a NAD-dependent protein lysine demalonylase and desuccinylase. *Science*.

[147] Rardin M. J., He W., Nishida Y. (2013). SIRT5 regulates the mitochondrial lysine succinylome and metabolic networks. *Cell Metabolism*.

[148] Lin Z.-F., Xu H.-B., Wang J.-Y. (2013). SIRT5 desuccinylates and activates SOD1 to eliminate ROS. *Biochemical and Biophysical Research Communications*.

[149] North B. J., Marshall B. L., Borra M. T., Denu J. M., Verdin E. (2003). The human Sir2 ortholog, SIRT2, is an NAD+-dependent tubulin deacetylase. *Molecular Cell*.

[150] Black J. C., Mosley A., Kitada T., Washburn M., Carey M. (2008). The SIRT2 deacetylase regulates autoacetylation of p300. *Molecular Cell*.

[151] Si X., Chen W., Guo X. (2013). Activation of GSK3*β* by Sirt2 is required for early lineage commitment of mouse embryonic stem cell. *PLoS ONE*.

[152] Ming M., Qiang L., Zhao B., He Y. Y. (2014). Mammalian SIRT2 inhibits keratin 19 expression and is a tumor suppressor in skin. *Experimental Dermatology*.

[153] Jing E., Gesta S., Kahn C. R. (2007). SIRT2 regulates adipocyte differentiation through FoxO1 acetylation/deacetylation. *Cell Metabolism*.

[154] Liu G.-Y., Jiang X.-X., Zhu X. (2015). ROS activates JNK-mediated autophagy to counteract apoptosis in mouse mesenchymal stem cells in vitro. *Acta Pharmacologica Sinica*.

[155] Inoue T., Nakayama Y., Li Y. (2014). SIRT2 knockdown increases basal autophagy and prevents postslippage death by abnormally prolonging the mitotic arrest that is induced by microtubule inhibitors. *FEBS Journal*.

[156] Zhao Y., Yang J., Liao W. (2010). Cytosolic FoxO1 is essential for the induction of autophagy and tumour suppressor activity. *Nature Cell Biology*.

